# Suboptimal Response to Eculizumab in Renal‐Limited Thrombotic Microangiopathy Associated With Malignant Hypertension and Monoclonal Gammopathy: A Case Report

**DOI:** 10.1002/ccr3.73712

**Published:** 2026-10-12

**Authors:** Wenjuan Lei, Tianli Shi, Man Xu, Jun Zhou

**Affiliations:** ^1^ Department of Nephrology Haikou People's Hospital Haikou China

**Keywords:** complement activation, eculizumab, malignant hypertension, monoclonal gammopathy, renal failure, thrombotic microangiopathy

## Abstract

Renal‐limited thrombotic microangiopathy (TMA) is clinically milder but etiologically complex and is often underdiagnosed. This case presents as complement‐mediated TMA with malignant hypertension and monoclonal gammopathy but showed suboptimal response to eculizumab treatment, suggesting heterogeneity in both etiology and treatment outcomes.

## 1 Introduction

Thrombotic microangiopathies (TMA) are typically characterized by microangiopathic hemolytic anemia, thrombocytopenia, and end‐organ ischemia [1]. However, they can also present as renal‐limited TMA (RL‐TMA) without systemic manifestations, caused by various etiologies, with complement activation being the underlying core mechanism. The treatment strategy involves correcting dysregulation of the complement system [2]. The complement component 5 (C5) inhibitor eculizumab has become a first‐line therapy for atypical hemolytic uremic syndrome (aHUS) [3], but clinical experience regarding its efficacy in RL‐TMA remains limited. This article reports a case of RL‐TMA with a suboptimal response to eculizumab, potentially due to delayed initiation of the C5 inhibitor and coexisting multifactorial pathogenesis.

## 2 Case History and Examination

A 37‐year‐old male presented to the Gastroenterology Department with intermittent melena. He had severe hypertension and renal failure (serum creatinine: 437 μmol/L). Following stabilization of gastrointestinal hemorrhage, the patient was transferred to our department for further management. The patient had a 5‐year history of hypertension, for which he had not received any antihypertensive medication. His grandfather and father were both diagnosed with hypertension in their 30s. Physical examination was largely unremarkable other than blood pressure (BP) was high (218/140 mmHg), pallor suggestive of anemia, and mild left cardiac border enlargement.

A laboratory data revealed the following: urinalysis showed proteinuria (2+), 24‐h urinary protein was 999 mg/day; serum creatinine level of 455 μmol/L, hemoglobin was 99 g/L; platelet count 578 × 10^9^/L, there were no schistocytes, with normal lactate dehydrogenase and bilirubin. All complement and immunologic parameters (Including anti‐topoisomerase I (Scl‐70) antibodies, and anti‐RNA polymerase III antibodies, among others.) were negative. Serum protein electrophoresis revealed a suspicious monoclonal protein band. Immunofixation electrophoresis demonstrated a suspicious IgA‐κ monoclonal immunoglobulin. Cardiac evaluation revealed hypertensive heart disease with left ventricular hypertrophy and diastolic dysfunction on echocardiography, accompanied by left ventricular high voltage and ST‐T changes on the electrocardiogram. Fundoscopy showed no acute hypertensive retinopathy. No renal artery stenosis was detected by doppler ultrasound. Gastroscopic findings revealed: (1) Chronic bile reflux gastritis; (2) Multiple gastric ulcers of unknown nature; (3) Multiple duodenal ulcers (Stage H1). Pathological examination showed: (gastric angle and gastric antrum biopsy tissues) mild glandular atypia, significant neutrophil infiltration in the stroma with necrosis present, and no obvious intestinal metaplasia. Results: (gastric angle) and (gastric antrum) chronic ulcers.

## 3 Differential Diagnosis, Investigations

This patient had a very broad differential diagnosis given his multiple complaints in the setting of uncontrolled hypertension with renal impairment, anemia, and monoclonal protein. Initially, there was suspicion for multiple myeloma and monoclonal gammopathy of renal significance (MGRS). The serum free light chain assay revealed an abnormal involved/uninvolved free light chain concentration (iFLC/uFLC) ratio of 2.73 (reference range: 0.26–1.65, Reference range for renal function correction: less than 3.1), with an involved free light chain concentration (iFLC) of 85.68 mg/L. The morphological results of the bone marrow aspiration biopsy demonstrated normocellular marrow with no evidence of clonal plasma cell proliferation.

After intensive antihypertensive treatment, BP was maintained at approximately 150/90 mmHg, and there was partial renal functional improvement. Renal biopsy was performed on the 45th day after onset; the patient ultimately agreed to proceed with the renal biopsy after thorough repeated recommendations. At this time, serum creatinine is 340 μmol/L. The kidney biopsy revealed that among the 12 glomeruli examined, three were globally sclerosed. One small fibrous‐crescent, mild mesangial hypercellularity and matrix expansion in remaining glomeruli, periglomerular fibrosis in some glomeruli, and ischemic collapse of capillary tufts. Chronic tubulointerstitial nephritis with severe tubular atrophy (~80%) and diffuse interstitial fibrosis. The arteriole wall is thickened with mucinous degeneration, showing an onion‐skin intimal hyperplasia, luminal stenosis, and visible thrombus formation. Direct immunofluorescence microscopy showed no deposits of immunoglobulins or complement. Electron microscopy reveals extensive foot process effacement with no definitive electron‐dense deposits. Immunoelectron microscopy shows no clear evidence of monoclonal light chain deposition (Figure 1). The findings were consistent with thrombotic microangiopathy associated renal injury, primarily involving renal arterioles. No evidence of direct renal injury attributable to monoclonal gammopathy was found on the kidney biopsy.

**FIGURE 1:**
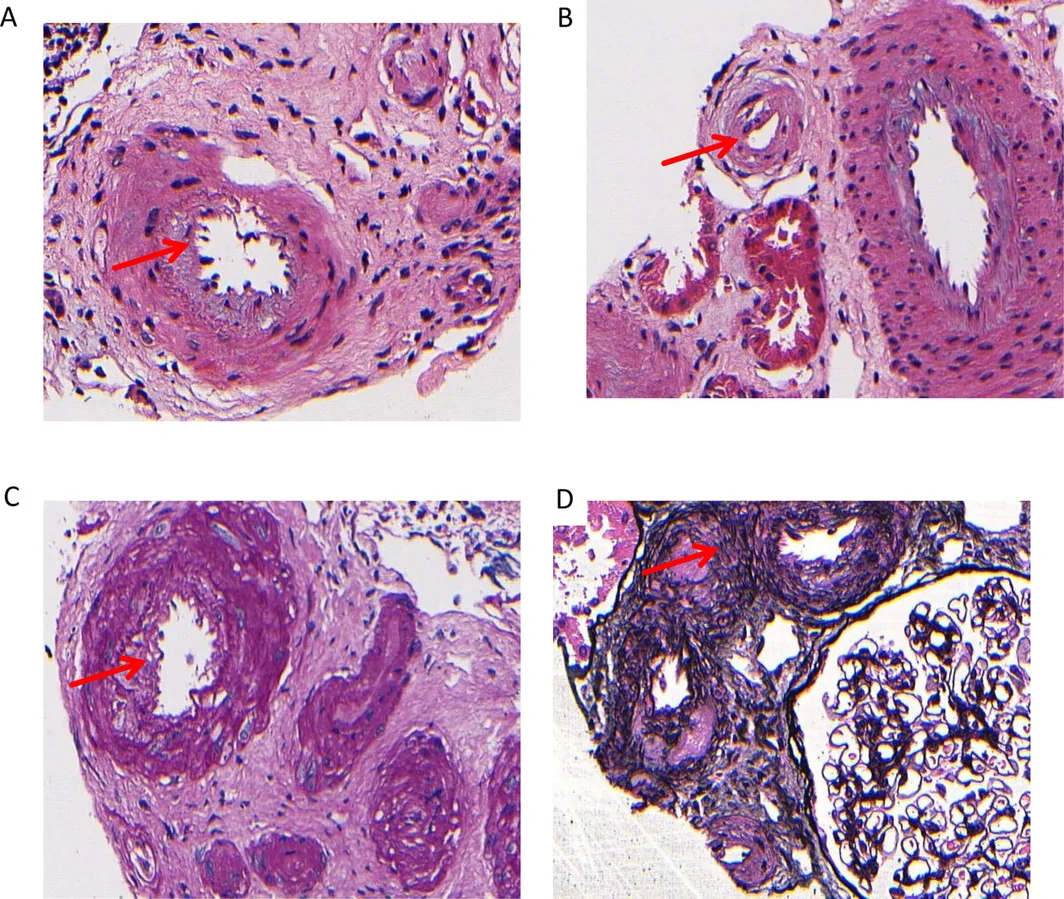
Renal biopsy findings. (A) Renal small artery intimal thickening with mucoid degeneration (arrow) was observed (Hematoxylin–Eosin staining × 400). (B) The small artery shows a thickened wall and narrowed lumen (arrow) (Hematoxylin–Eosin staining × 400). (C) The vascular intima is thickened with luminal stenosis (arrow) (Periodic Acid‐Schiff × 400). (D) The vascular lumen contains thrombus formation (arrow) (Periodic Acid‐Silver Methenamine × 400).

This patient had no systemic hematologic involvement, consistent with localized renal TMA. RL‐TMA may arise from diverse underlying causes, including aHUS, drugs, malignant tumors, autoimmune diseases, MGRS, etc. except for thrombotic thrombocytopenic purpura and *streptococcus pneumoniae* . Further complement‐related workup revealed that soluble membrane attack complex (sC5b‐9) significantly elevated at > 2100 ng/mL (normal range: 75–219 ng/mL). No detectable abnormalities in human complement factors H, B, or I antibodies, ADAMTS13 activity and ADAMTS13 inhibitory antibodies. Whole‐exome sequencing showed that no pathogenic or likely pathogenic variants in genes associated with the complement pathway; likewise, no variants of uncertain clinical significance (VUS) were identified. Stool culture was negative. The lack of findings that Shiga toxin‐producing *Escherichia coli* infection‐mediated HUS and thrombotic thrombocytopenic purpura indicated complement‐mediated TMA.

## 4 Conclusion and Results

After intensifying the antihypertensive regimen with sacubitril/valsartan, we started anti‐C5 monoclonal antibody treatment (eculizumab was initiated at 900 mg weekly for 4 weeks, followed by 1200 mg every 2 weeks). Serial monitoring showed serum creatinine initially decreased from 277 μmol/L to 248 μmol/L, but subsequently rebounded to 335 μmol/L by Week 10 of therapy, suggesting incomplete treatment response. Eculizumab was therefore discontinued. During treatment, the sC5b‐9 level ranged from 280 to 335 ng/mL, with a 50% hemolytic complement (CH50) value of 13.7–13.8 U/mL (reference range: 23.0–46.0 U/mL), indicating complete and sustained inhibition of the terminal complement pathway. The patient maintained stable blood pressure control without infectious complications. Repeat testing confirmed persistent IgA‐kappa monoclonal gammopathy. Following hematology consultation, a diagnosis of monoclonal gammopathy of renal significance was suspected. Unfortunately, however, the patient refused a repeat kidney biopsy for further clarification. After obtaining the patient's consent, anti‐myeloma therapy was initiated the day after eculizumab discontinuation, consisting of weekly bortezomib 2.6 mg/m^2^, cyclophosphamide 600 mg, and dexamethasone 20 mg. Following the treatment adjustment, serum creatinine levels showed a gradual decline. At Week 7 of anti‐myeloma therapy, serum creatinine had decreased to 261 μmol/L. The patient declined further treatment for personal reasons. At the follow‐up visit on Day 300, serum creatinine was 233 μmol/L, and the monoclonal protein persisted at low concentration (iFLC: 36.82 mg/L; iFLC/uFLC ratio: 1.91). Trends in lab parameters in relation to the timing of intervention are shown in Figure 2.

**FIGURE 2:**
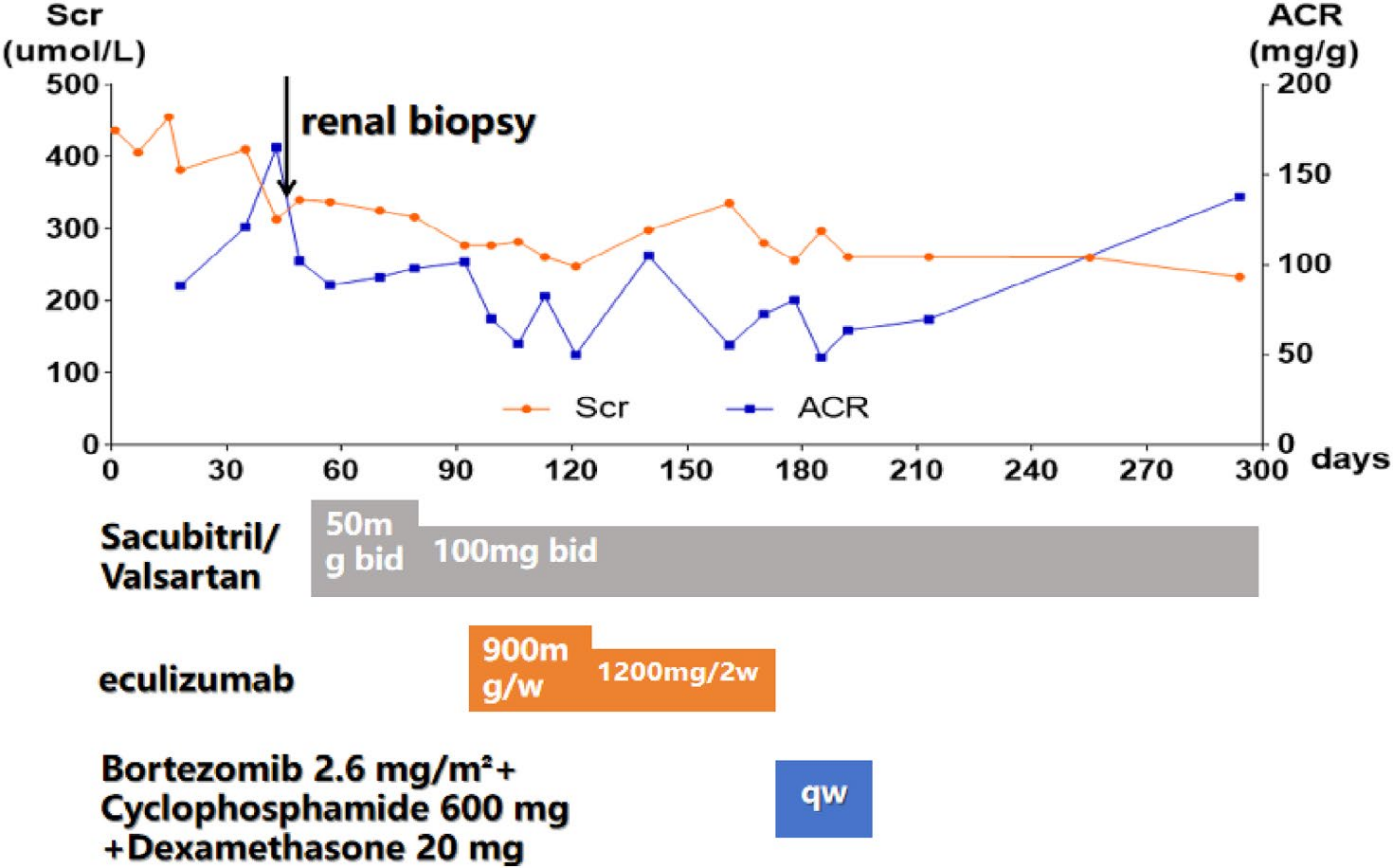
Clinical and therapeutic course. Scr, serum creatinine; ACR, Urine protein/creatinine ratio.

## 5 Discussion

TMA is a pathological disorder triggered by endothelial injury and/or dysfunction, manifesting as various disease entities. It is characterized by thrombocytopenia, microangiopathic hemolytic anemia (MAHA), and end‐organ damage, with acute kidney injury (AKI) being a prominent feature due to the pronounced susceptibility of glomerular circulation to endothelial damage and occlusion [1]. TMA should be suspected when hemolytic anemia and organ dysfunction are present. However, MAHA and thrombocytopenia are not absolute diagnostic requirements for TMA. With increasing clinical observations and advances in understanding its etiology and pathogenesis, the spectrum of TMA has broadened. TMA no longer exclusively presents with microangiopathic hemolysis. In clinical practice, many cases present with isolated elevated serum creatinine, without systemic involvement. Kidney biopsy confirms TMA pathology, defining these cases as RL‐TMA. A French retrospective study found that RL‐TMA accounts for up to 45% [2]. However, RL‐TMA's indolent clinical course with preserved renal function, typically baseline serum creatinine < 270 μmol/L, and lack of dramatic hematologic manifestations lead to frequent diagnostic oversight.

This article describes a young male patient presenting with renal function and malignant hypertension; renal biopsy demonstrated TMA. It represents a classic RL‐TMA, which would have been missed without kidney biopsy due to lacking classic hematologic TMA features and non‐specific clinical presentation. This patient presented with gastrointestinal bleeding. Given the confirmed diagnosis of RL‐TMA, the gastrointestinal tract, as a potential target organ of microvascular thrombosis, warranted further investigation for gastrointestinal TMA. Gastroscopy revealed multiple active lesions, including gastric angle ulcers and duodenal bulb ulcers. Further biopsy of the lesions showed chronic inflammatory changes with granulation tissue formation and focal intestinal metaplasia, without microvascular injury features such as intravascular thrombi, endothelial cell swelling, or fibrinoid necrosis, which did not support a diagnosis of gastrointestinal TMA.

RL‐TMA may arise from diverse underlying causes, encompassing virtually all etiologies associated with systemic TMA, including aHUS, drugs, malignant tumors, autoimmune diseases, infection, pregnancy, etc. except for thrombotic thrombocytopenic purpura and *streptococcus pneumoniae* [3]. Elias et al. [4] concluded that the most common causes of RL‐TMA were drugs (28.5%) and underlying rheumatic diseases (20%). Maisons et al. identified aHUS as the most common cause of RL‐TMA, with complement‐mediated aHUS being 25% of the causes [2]. The patient's serum sC5b‐9 > 2100 ng/mL suggests complement‐mediated TMA.

Many patients with TMA have multiple etiologies, making it difficult to distinguish between causes and triggers, especially when the etiologies involve malignancy, drug‐induced, malignant hypertension, and autoimmune diseases, where there is a large overlap of causes or triggers. In our case, while the precise etiology remains undetermined, malignant hypertension and monoclonal gammopathy were considered the most probable contributors. The renal pathology demonstrating predominant arteriolar involvement, along with partial renal function recovery after blood pressure control and eculizumab therapy, supports hypertensive nephropathy as one mechanism. However, the serum creatinine rebounded at week 10 of complement inhibition. The absence of demonstrable monoclonal protein deposition on histopathology does not definitively rule out paraprotein induced complement activation through indirect pathways. The lack of follow‐up biopsy during renal function deterioration represents a significant diagnostic limitation. Notably, the patient's IgA‐κ paraprotein prompted empirical plasma cell directed therapy with bortezomib, dexamethasone, and cyclophosphamide. The subsequent creatinine improvement implicates monoclonal immunoglobulin mediated renal injury as another pathogenic mechanism in this complex TMA phenotype. Ravindran et al. analyzed 146 TMA patients at the Mayo Clinic and found that 13.7% had paraproteinemia, which is four times more common in TMA patients, especially those with localized renal TMA, as a potential susceptibility factor for the “second hit” of TMA [5], which plays a potential role in the development of TMA [6]. Therefore, patients with TMA should be routinely screened for underlying monoclonal immunoglobulinemia.

C5 inhibitor is the first‐line treatment of aHUS. Eculizumab, a recombinant humanized monoclonal antibody against C5, inhibits the formation of the membrane attack complex, thereby suppressing complement activation. Approved by the FDA in 2011 for the treatment of atypical hemolytic uremic syndrome. Prompt initiation of C5 blockade is critical for both short‐term and long‐term outcomes [7, 8], and it is believed that eculizumab therapy should be initiated within 24 ~48 h of onset to control disease progression [9, 10, 11]. The diagnosis of RL‐TMA remains clinically challenging due to the absence of specific laboratory biomarkers, with confirmation typically requiring renal biopsy. This diagnostic difficulty often leads to delays in initiating targeted therapy. Currently, C5 inhibitor therapy is rarely used for RL‐TMA, and its efficacy requires further evaluation. Research on RL‐TMA remains extremely limited. To date, a PubMed search using the combined terms “thrombotic microangiopathy” and “renal‐limited” yields only 22 references, including six review articles. Among these, only four publications involving a total of 11 cases specifically address eculizumab treatment [2, 6, 12, 13] (Table 1). The affected population predominantly consists of middle‐aged individuals, with a higher prevalence in males than females. The etiologies varied across cases. Following eculizumab treatment, 2 patients responded effectively with renal function recovery and discontinuation of dialysis, while 5 patients showed no improvement and progressed to end‐stage renal disease. These case observations suggest that eculizumab demonstrates inferior efficacy in RL‐TMA compared to systemic TMA.

**TABLE 1:** Summary of case reports on eculizumab treatment for renal‐limited TMA.

Author	Number of examples	Gender/age	Pathogen	Pre‐treatment sC5b‐9 (ng/mL)	Pre‐treatment serum creatinine (μmol/L)	Renal outcome (μmol/L)	Follow‐up duration (month)
Maisons et al. [2] 2023	6	NA	aHUS	NA	402	50% ESRD	70
Martins et al. [6] 2022	1	M/58	Monoclonal protein (IgG‐λ)	596	Hemodialysis	Discontinuation of dialysis	17
Bharati et al. [12] 2023	1	M/58	Medications (tacrolimus, everolimus)	374	283	Maintenance dialysis	NA
Sjoerd et al. [13] 2018	3	M/47	Malignant hypertension	NA	1980	297	5
		F/44	Malignant hypertension	3800	649	ESRD	20
		F/41	Malignant hypertension	4200	334	315	32
This article	1	M/37	Malignant hypertension, monoclonal protein (IgA‐k)	> 2100	277	335	11

In our case, the patient received a 10‐week regimen of 900 mg weekly for 4 weeks followed by 1200 mg every 2 weeks for three doses. While serum creatinine decreased to 248 μmol/L during the first month of treatment, it subsequently rebounded to 335 μmol/L for unclear reasons. This case is similar to the cases with poor renal prognosis in studies by Maisons et al. [2] and Sjoerd et al. [13]. According to international studies, 57.1% of patients showed improvement in serum creatinine levels after 1 month of eculizumab treatment [14]. Considering this patient's suboptimal therapeutic response to eculizumab therapy, the treatment was discontinued. After eculizumab therapy, serum creatinine decreased from its baseline level, indicating a partial treatment response; however, it did not return to normal. This outcome is likely attributable to the delayed initiation of complement inhibition. Renal biopsy revealed approximately 80% tubular atrophy with diffuse interstitial fibrosis—structural changes that are irreversible despite complement blockade. During the course of treatment, serum creatinine rebounded. Although CH50 remained consistently fully suppressed and sC5b‐9 levels significantly decreased to near‐normal ranges—confirming effective complement inhibition—renal function may still deteriorate in some patients due to complement‐independent progressive mechanisms.

Immunofixation electrophoresis persistently detected an IgA‐κ monoclonal protein in this patient. Although bone marrow morphology revealed no evidence of clonal plasma cell proliferation, a monoclonal plasma cell disorder could not be definitively excluded, as the patient declined further diagnostic evaluation—including bone marrow flow cytometry, immunophenotyping, and repeat renal biopsy—citing financial constraints. A hematology consultation suggested that the monoclonal immunoglobulin may serve as a potential trigger for the ongoing renal injury, possibly contributing to sustained endothelial damage through complement‐independent mechanisms. Despite repeated discussions with the patient and their family regarding the necessity of additional workup, they ultimately declined further testing and requested diagnostic therapy instead. Based on the IgA‐κ monoclonal protein profile, plasma cell‐directed therapy was initiated with bortezomib 2.6 mg/m^2^ + dexamethasone 20 mg + cyclophosphamide 600 mg weekly. By Week 7 of therapy, serum creatinine levels decreased to 261 μmol/L. At the follow‐up visit on Day 300, serum creatinine was 233 μmol/L, the monoclonal protein persists at low concentration (iFLC: 36.82 mg/L; iFLC/uFLC ratio: 1.91). After anti‐plasma cell therapy, the patient's renal function showed sustained improvement, accompanied by a concurrent decline in monoclonal protein levels. This provides compelling clinical evidence supporting monoclonal immunoglobulin as a potential underlying cause of RL‐TMA.

In summary, this report describes an uncommon case of renal‐limited thrombotic microangiopathy (RL‐TMA) in a 37‐year‐old male who presented with acute kidney injury but lacked the classic hematologic features of systemic TMA. The clinical phenotype was characterized by complement‐mediated TMA associated with malignant hypertension and monoclonal gammopathy, exhibiting a suboptimal response to eculizumab despite complete and sustained inhibition of the terminal complement pathway. The subsequent improvement in renal function following plasma cell‐directed therapy highlights the multifactorial pathogenesis underlying this complex TMA phenotype. These findings underscore the importance of routine screening for underlying monoclonal immunoglobulinemia in patients with TMA and the prompt initiation of C5 inhibitor therapy to optimize treatment outcomes.

## Author Contributions

**Wenjuan Lei:** conceptualization, writing – original draft, writing – review and editing. **Tianli Shi:** data curation, writing – review and editing. **Man Xu:** resources, writing – review and editing. **Jun Zhou:** conceptualization, formal analysis, supervision, writing – review and editing.

## Funding

This work was supported by the Hainan Provincial Natural Science Foundation of China [grant numbers 825MS184] and Hainan Provincial Joint Project on Health Science and Technology Innovation [grant numbers WSJK2026QN157].

## Consent

We obtained written informed consent from the patient for the publication of this case. The patient himself signed the consent form with his full legal name.

## Conflicts of Interest

The authors declare no conflicts of interest.

## Data Availability

The data that support the findings of this study are available from the corresponding author upon reasonable request.
